# Mental Health Consequences of the COVID-19 Pandemic Among Older Adults With Chronic Conditions

**DOI:** 10.1093/geroni/igab046.2681

**Published:** 2021-12-17

**Authors:** Courtney Polenick, Diarratou Kaba, Summer Meyers, Annie Zhou, Nikita Daniel, Shreya Salwi, Vanessa Lee

**Affiliations:** 1 University of Michigan, Ann Arbor, Michigan, United States; 2 University of Michigan, University of Michigan, Michigan, United States

## Abstract

The COVID-19 pandemic may have a negative impact on mental health, especially among older adults with chronic conditions who are more vulnerable to severe illness. This cross-sectional qualitative study evaluated how the pandemic has impacted the ways that adults aged 50 and older with chronic conditions managed their mental health. Participants included a total of 492 adults (M = 64.95 years, SD = 8.91, range = 50 – 94) from Michigan (82.1%) and 33 other U.S. states who reported a diagnosis of at least one chronic condition and completed an anonymous online survey between May 14 and July 9, 2020. Participants provided open-ended responses to a question about the pandemic’s impact on how they were taking care of their mental health. The data were coded to ascertain relevant concepts and were reduced to develop major themes. We determined four main themes. The pandemic impacted how participants took care of their mental health through: (1) pandemic-related barriers to social interaction; (2) pandemic-related routine changes; (3) pandemic-related stress; and (4) pandemic-related changes to mental health care. Taken as a whole, this study indicates that older adults with chronic conditions encountered a variety of challenges to managing their mental health in the early months of the COVID-19 pandemic, but also demonstrated considerable resilience. These findings identify potential risk and protective factors to target as part of personalized interventions to preserve their well-being during this pandemic and in future public health crises.

